# *Lactobacillus reuteri* DSM 17938 and ATCC PTA 5289 ameliorates chemotherapy-induced oral mucositis

**DOI:** 10.1038/s41598-020-73292-w

**Published:** 2020-10-01

**Authors:** Nitasha Gupta, Joao Ferreira, Catherine Hsu Ling Hong, Kai Soo Tan

**Affiliations:** 1 Faculty of Dentistry, National University of Singapore, 9 Lower Kent Ridge Road, Singapore, 119085 Singapore; 2 Exocrine Gland Biology and Regeneration Research Group, Faculty of Dentistry, Chulalongkorn University, Bangkok, Thailand

**Keywords:** Microbiology, Diseases

## Abstract

Oral mucositis (OM) is a common complication of cancer therapy, however OM management remains unsatisfactory. There is a growing interest in the therapeutic potential of probiotics in OM due to positive findings of its use in intestinal mucositis. This study aimed to determine the efficacy and safety of the probiotic combination *Lactobacillus reuteri* DSM 17938 and ATCC PTA 5289 strains in chemotherapy-induced OM. Mice were divided into 4 groups. PBS/water and PBS/LR groups comprised of mice injected with PBS intraperitoneally (*i.p.*)*,* and were given water or the mixture of *L. reuteri* (LR) DSM 17938 and ATCC PTA 5289 in water respectively. The 5-FU/water and 5-FU/LR groups comprised of mice injected with 5-FU *i.p.*, and were given water or *L. reuteri* DSM 17938 and ATCC PTA 5289 in water respectively. Histopathological analysis revealed that the oral epithelia of the 5-FU/water and 5-FU/LR groups were thinner compared to PBS/water and PBS/LR groups. However, epithelial damage was significantly reduced in the 5-FU/LR compared to 5-FU/water group. Additionally, the 5-FU/LR group showed reduced oxidative stress and inflammation in the oral mucosa. We further showed that *L. reuteri* reduced oxidative stress through the nuclear factor E2-related factor-2 (Nrf-2) signalling. There was no evidence of translocation of *L. reuteri* systemically. This study demonstrated for the first time that *L. reuteri* protected oral mucosa against damage induced by chemotherapy.

## Introduction

Anti-neoplastic therapy induced oral mucositis (OM) is a painful condition affecting patients’ ability to eat, drink, swallow and speak^1^. Those suffering from severe OM may require parenteral nutrition, intravenous analgesics, extended hospitalizations which can result in the disruption of cancer treatment and morbidity. The prevalence of OM is high, affecting up to 40% and 100% of patients undergoing chemo- and head and neck radiotherapy respectively^2^.

OM is a consequence of both direct and indirect damage to the oral mucosa following anti-neoplastic therapy. These therapies damage DNA and trigger the production of reactive oxygen species (ROS). During the initiation phase of OM, ROS induces the activation of nuclear factor kappa B (NFκB) which is a major signalling pathway whereby cytokines such as interleukin-1β (IL-1β), interleukin-6 (IL-6) and tumour necrosis factor-α (TNF-α) are produced^3,4^. These pro-inflammatory mediators amplify the initial immune response inducing a state of hyper-inflammation. Additionally, danger associated molecular patterns (DAMPs) such as high-mobility group box-1 (HMGB-1) released from damaged or dead cells further exacerbate the inflammatory response^5^. The accumulation of these inflammatory responses ultimately lead to marked destruction of the oral epithelium and ulceration of the oral mucosa, a hallmark of OM^4,6^.

There is emerging evidence on the role of microbiota in the pathogenesis of OM. In animal studies, higher bacterial loads was associated with greater OM severity^7^. However, these observations may be causal or consequential. A recent proof of concept study demonstrated that conventional animals suffered worse OM compared to their germ-free counterparts, providing evidence of the detrimental impact of oral microbiota on the severity of OM^8^. In clinical studies, a shift from a largely Gram positive to Gram negative oral microbiota during cancer therapy has been reported^9^. This is a significant event as the increased colonization of the damaged mucosa by Gram negative microorganisms could intensify the inflammatory responses which worsens OM through the production of bacterial endotoxins^10^.

Although, OM has been reported by patients to be the most debilitating complication of cancer therapy, OM management remains unsatisfactory. To reduce the oral bacterial load and to prevent secondary infections, the use of systemic or topical antimicrobial agents have been investigated^11,12^. However, these approaches have yielded inconsistent clinical benefits suggesting that the indiscriminate reduction of oral microbiota is not a panacea for the management of OM. Instead, a therapeutic approach targeting both oral microbiota dysbiosis and host immune response may yield greater clinical benefits.

Probiotics are defined as “live micro-organisms which when administered in adequate amounts, confer a health benefit on the host”. *Lactobacillus* spp. is one of the dominant generas of probiotics, comprising of *L. acidophilius, L. rhamnosus, L. casei* and *L. reuteri*. *L. reuteri* are natural inhabitants of the human body and can be found in the gastrointestinal tract, vagina and breast milk. *L. reuteri* DSM 17938 has been reported to alleviate chronic inflammatory conditions such as infantile colic and irritable bowel syndrome^13^. In oral inflammatory diseases, the administration of *L. reuteri* DSM 17938 and ATCC PTA 5289 have been associated with reduced gum inflammation, and a decrease in pathogens associated with periodontitis; suggesting the ability of these *L. reuteri* strains to modulate host inflammatory response, displace pathogenic bacteria and prevent dysbiosis by maintaining host-microbiome balance in the oral milieu^14,15^. Currently, the safety and efficacy of *L. reuteri* in OM is unknown. This study aimed to (1) evaluate the efficacy of *L. reuteri* DSM 17938 and ATCC PTA 5289 strains in reducing the severity of chemotherapy-induced OM in vivo, and (2) assess the safety of *L. reuteri* DSM 17938 and ATCC PTA 5289 administration by evaluating its potential translocation to the blood, spleen and liver.

## Results

### *Lactobacillus reuteri* (LR) DSM 17938 and ATCC PTA 5289 reduces the severity of chemotherapy-induced OM

**Figure 1:**
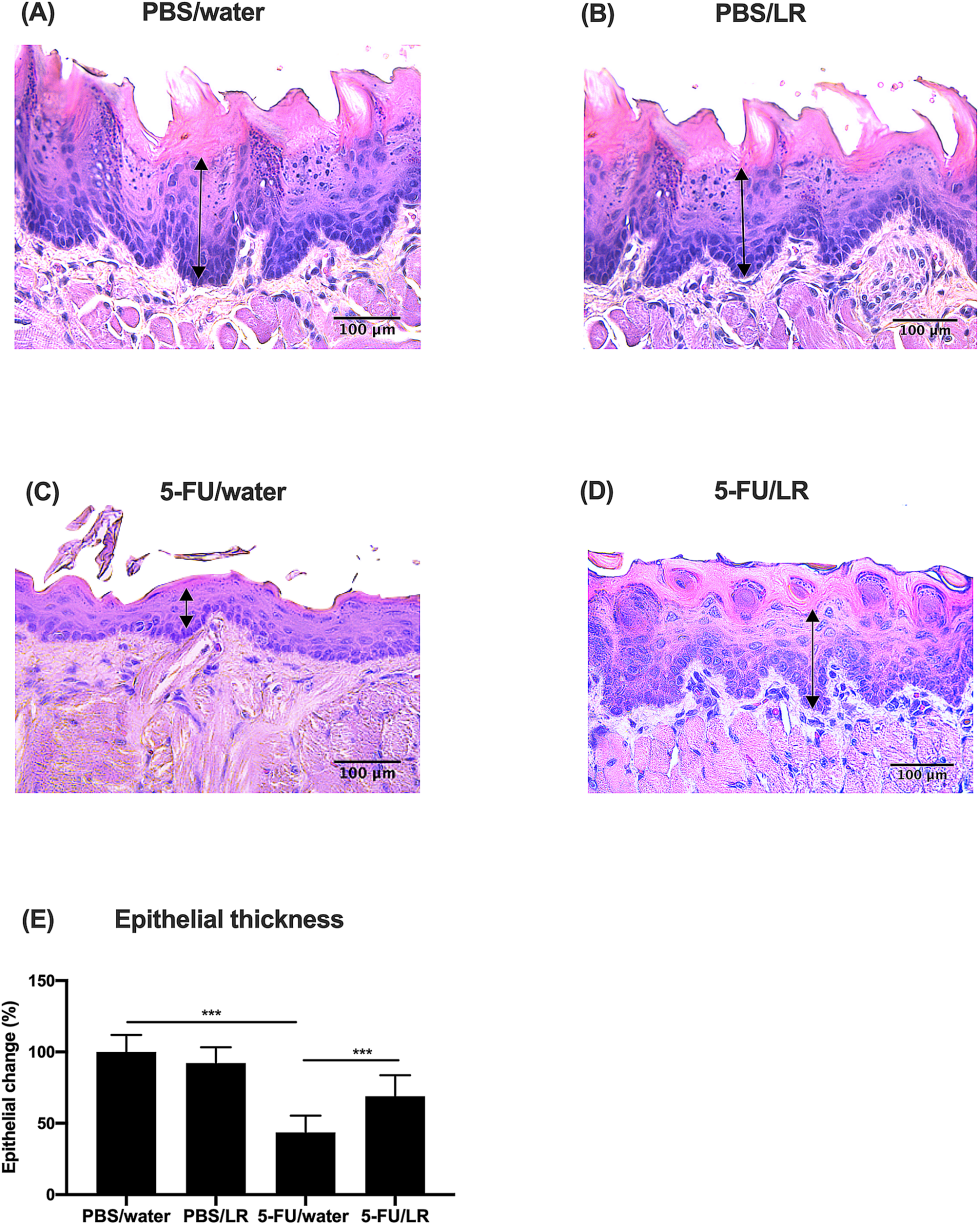
Effects of the mixture of *L. reuteri* (LR) strains on 5-FU-induced oral mucosa destruction. (**A**–**E**) Histopathological analysis (magnification x 400) of oral mucosa of mice. (**A**) PBS *i.p.* and fed with normal drinking water (PBS/water), (**B**) PBS *i.p.* and fed with LR in drinking water (PBS/LR), (**C**) 5-FU *i.p.* and fed with normal drinking water (5-FU/water) and (**D**) 5-FU *i.p.* and fed with LR in drinking water (5-FU/LR). Arrows indicate thickness of the oral epithelium. (**E**) Quantitative analysis of the thickness of the oral epithelium. ***p < 0.001*.*

**Figure 2:**
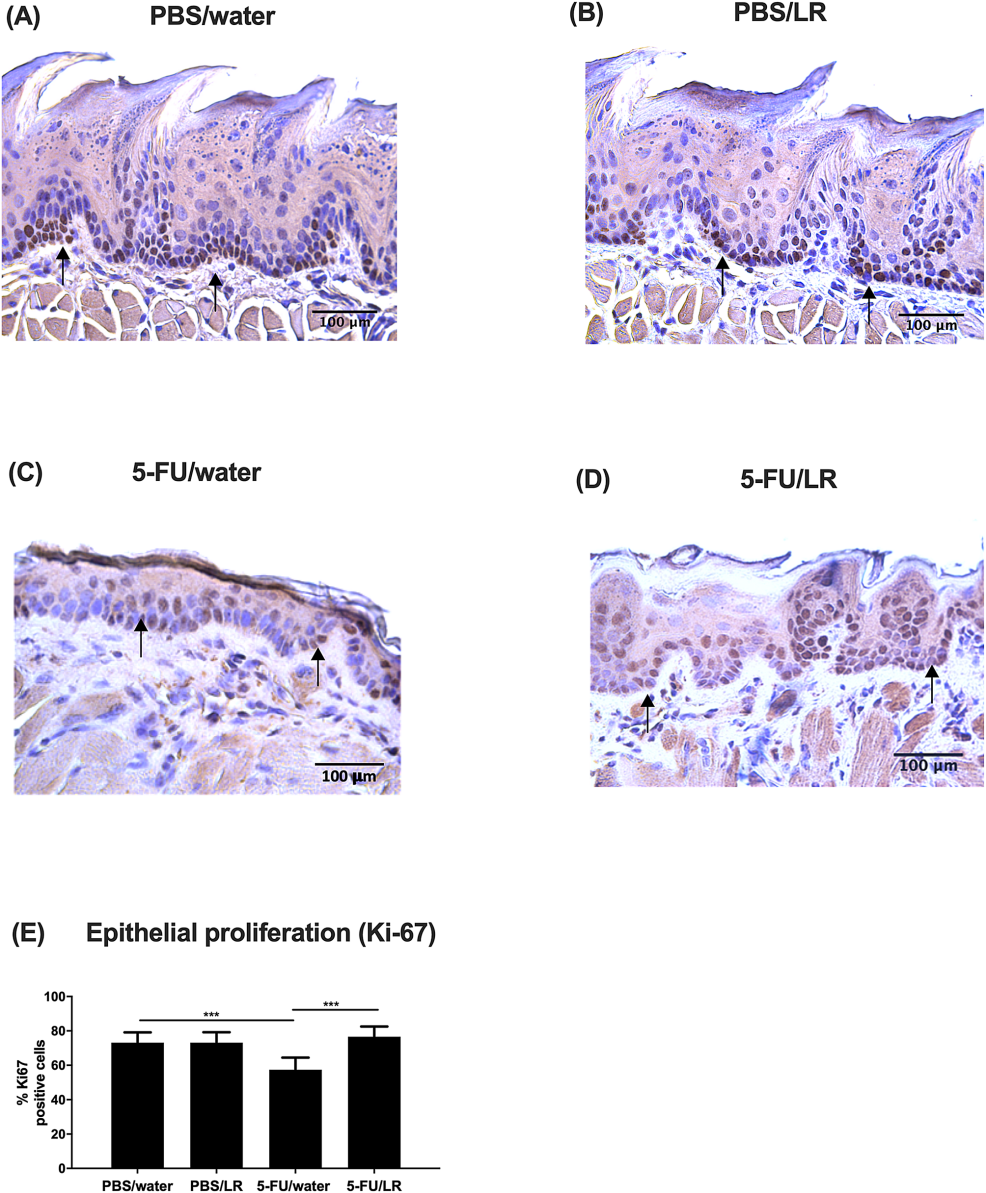
Effects of *L. reuteri* (LR) mixture on Ki-67 expression of oral mucosa. (**A**–**D**) Immunohistochemical analysis (magnification x 400) of the expression of Ki-67 in the basal epithelial of oral mucosa. (**A**) PBS *i.p.* with normal drinking water (PBS/water), (**B**) PBS *i.p.* and LR in drinking water (PBS/LR), (C) 5-FU *i.p.* with normal drinking water (5-FU/water) and (**D**) 5-FU *i.p.* with LR in drinking water (5-FU/LR). (**E**) Amount of Ki-67-stained positive cells. ***p < 0.001*.*

Histopathological analysis revealed that the oral epithelia of mice in the 5-FU/water and 5-FU/LR groups were thinner compared to those in the PBS/water and PBS/LR groups (Fig. 1). However, epithelial damage were less severe in the 5-FU/LR group compared to the 5-FU/water group (Fig. 1C,D). The quantitative analysis of the oral epithelium thickness confirmed the qualitative observations. Compared to the PBS/water and PBS/LR groups, the damage to the oral epithelia of the mice given the *L. reuteri* strains (5-FU/LR) was less compared to mice not given *L. reuteri* (5-FU/water) (Fig. 1E). Consistent with these histological observations, the expression of Ki-67 in the basal epithelial cells of the 5-FU/LR group was significantly higher compared to those in the 5-FU/water group (Fig. 2). No bacterial growth was found in blood, spleen and liver homogenates in all animals indicating that the *L. reuteri* strains used were safe for administration.

### *Lactobacillus reuteri* stimulates anti-inflammatory effects and increases resistance to oxidative stress

**Figure 3:**
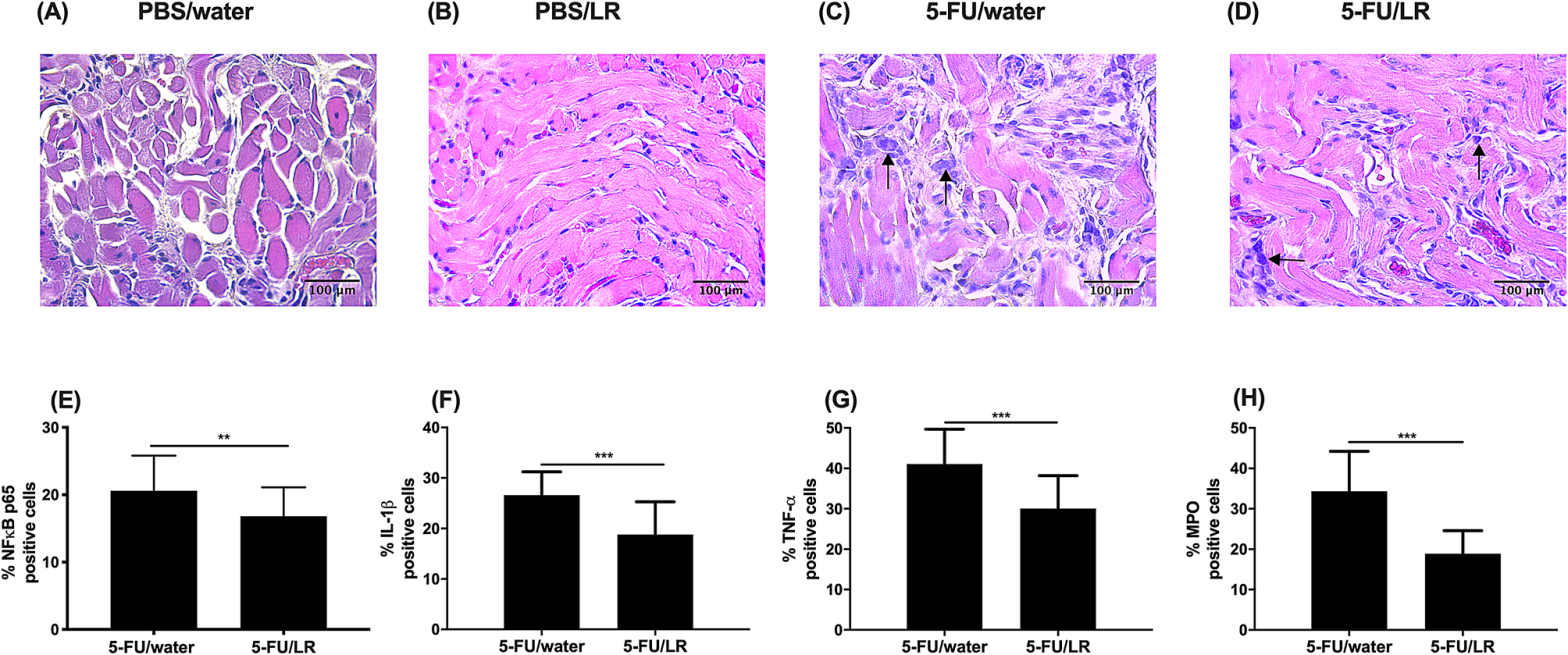
Effects of *L. reuteri* (LR) mixture on 5-FU-induced inflammation. (**A**–**D**) Histopathological analysis (magnification x 400) of the oral mucosa of mice. (**A**) PBS *i.p.* with normal drinking water (PBS/water), (**B**) PBS *i.p.* and LR in drinking water (PBS/LR), (**C**) 5-FU *i.p.* with normal drinking water (5-FU/water), (**D**) 5-FU *i.p.* with LR in drinking water (5-FU/LR). Arrows indicate inflammatory cells. (**E**–**H**) Immunohistochemical analysis of the expression of pro-inflammatory mediators in the oral mucosa of mice. (**E**) nuclear NFκB p65, (**F**) IL-1β, (**G**) TNF-α, and (**H**) MPO. **p < 0.01; ***p < 0.001*.*

**Figure 4:**
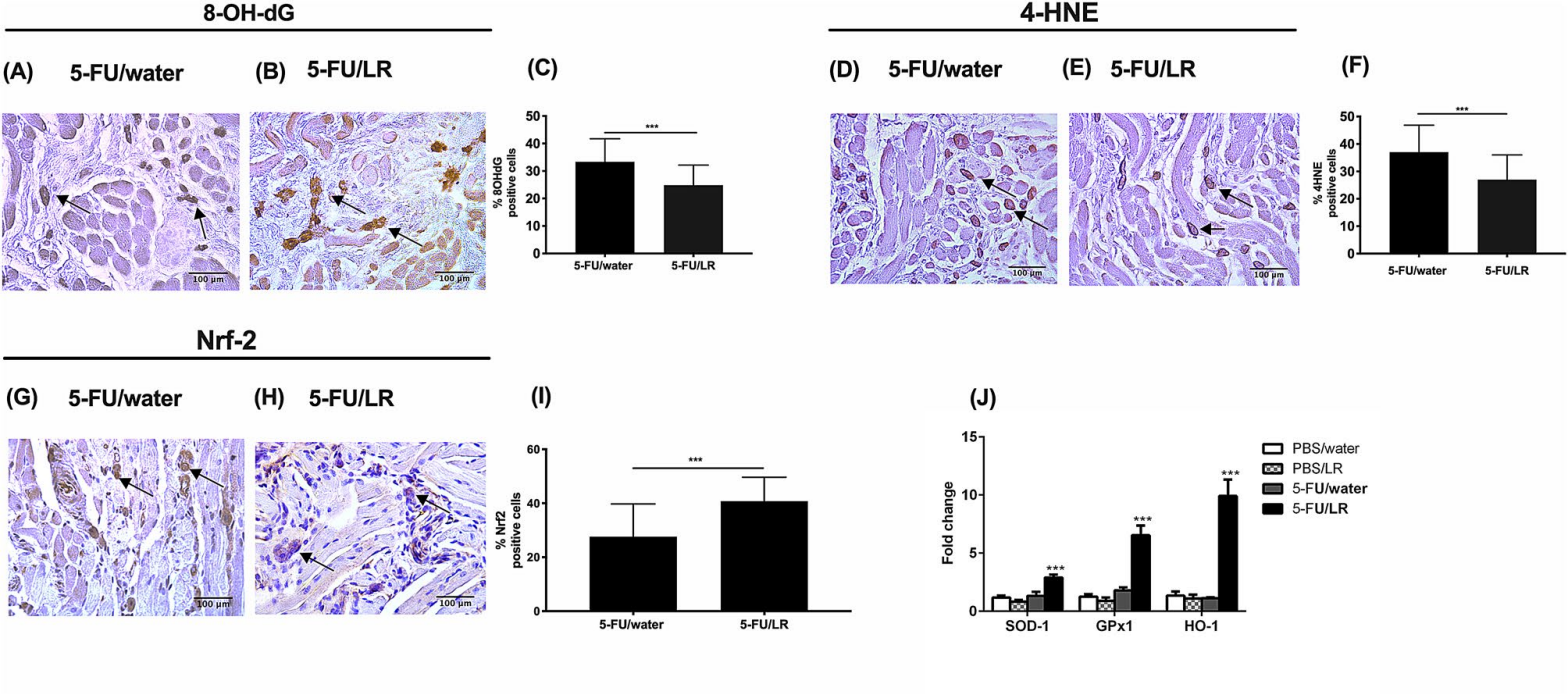
Effects of *L. reuteri* (LR) mixture on 5-FU-induced oxidative stress. Immunohistochemical analysis of the expression of (**A**,**B**) 8-OH-dG, (**D**,**E**) 4-HNE and (**G**,**H**) Nrf-2 in the oral mucosa of (**A**,**D**,**G**) 5-FU/water and (**B**,**E**,**H**) 5-FU/LR treated mice. Arrow indicates positively stained cells. Quantitative analysis of number of positively stained (**C**) 8-OH-dG, (**F**) 4-HNE, (**I**) Nrf-2 cells. (**J**) RT-qPCR analysis of antioxidant gene expression in the oral mucosa. ***p < 0.001*.*

Mice inoculated with 5-FU demonstrated increased accumulation of inflammatory infiltrates in the oral mucosa (Fig. 3). However, the amount of immune cell infiltration was less intense in the 5-FU/LR group compared to the 5-FU/water group (Fig. 3C,D). Aligned with these observations, the expression of pro-inflammatory mediators i.e., nuclear translocated NFκB p65, IL-1β, TNF-α and MPO were higher in the 5-FU/water group compared to the 5-FU/LR group; indicating that the *L. reuteri* strains exerted anti-inflammatory effects (Fig. 3E–H). In addition, the expressions of oxidative stress markers i.e. 8-hydroxy-2′-deoxyguanosine (8-OH-dG), a marker of DNA damage, and 4-hydroxynonenal (4-HNE), a marker of lipid peroxidation, were more pronounced in the 5-FU/water group compared to the 5-FU/LR group (Fig. 4A–F). Interestingly, the expression of nuclear factor E2-related factor-2 (Nrf-2), a key transcription factor regulating the expression of cytoprotective genes important in mitigating oxidative stress^16,17^, was significantly higher in the 5-FU/LR group compared to the 5-FU/water group (Fig. 4G–I). Consistent with these results, the expressions of key anti-oxidant genes, i.e. superoxide dismutase-1 (SOD-1), glutathione peroxidase-1 (GPx-1) and heme oxygenase-1 (HO-1), were greater in the oral mucosa of the 5-FU/LR group compared to the 5-FU/water group (Fig. 4J).

### *Lactobacillus reuteri* mediate cytoprotection via Nrf-2

**Figure 5:**
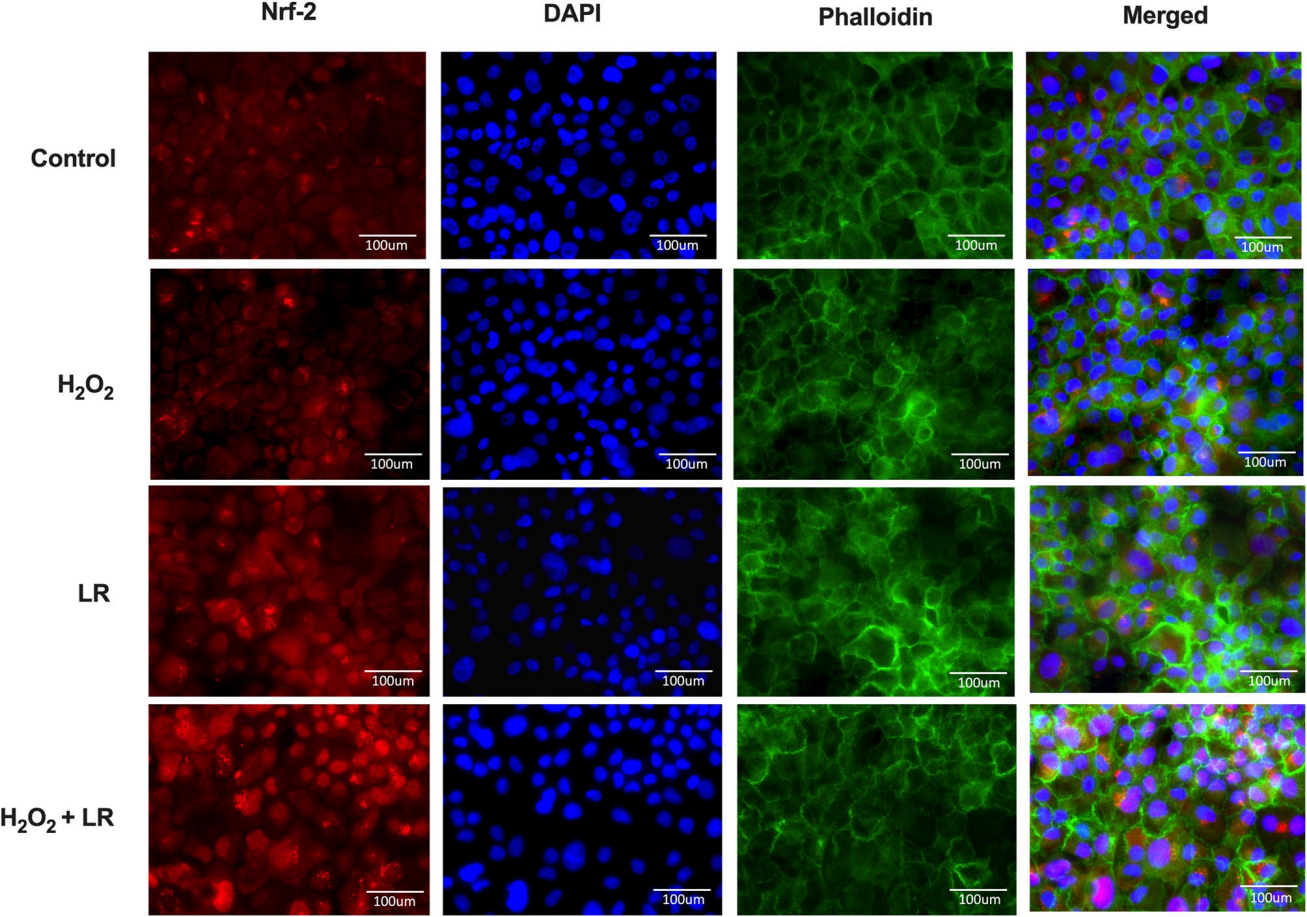
*Lactobacillus reuteri* (LR) mixture mitigates oxidative stress via Nrf-2. TR146 cells were either untreated (control) or pre-treated with LR DSM 17938 and ATCC PTA 5289 at a MOI of 1:1 for 1 h prior to treatment with 50 μM H_2_O_2_ for 3 h. The cellular location of Nrf-2 was determined by immunofluorescence. Nrf-2 was stained red, DAPI stained the nucleus blue while phalloidin stained the actin green. × 400 magnification. Representative immunofluorescence images are shown.

**Figure 6:**
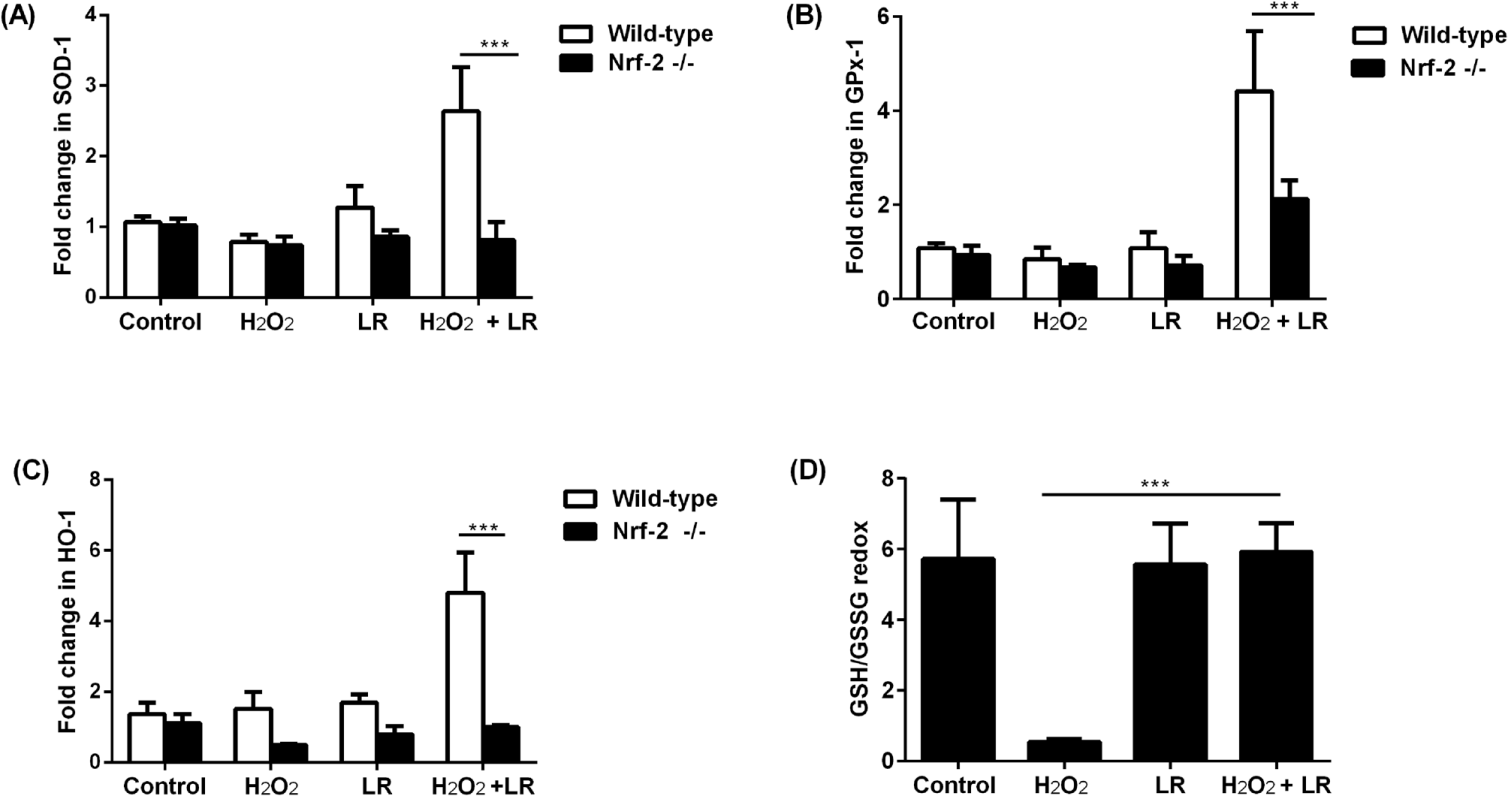
*Lactobacillus reuteri* (LR) mixture mitigates oxidative stress via Nrf-2. TR146 cells were either untreated (control) or pretreated with LR at a MOI of 1:1 for 1 h prior to treatment with 50 μM H2O2 for 3 h. The expression of (**A**) SOD-1, (**B**) GPx-1 and (**C**) HO-1 in wild-type and Nrf-2 null cells were determined by qPCR. (**D**) The concentrations of cellular GSH and GSSG were determined and expressed as a ratio. ***p < 0.001*.*

Using the TR146 oral mucosa cell line, it was observed that Nrf-2 was predominantly located in the cytoplasm in the control untreated cells (Fig. 5). TR146 cells were treated with hydrogen peroxide (H_2_O_2_,) at a non-cytotoxic concentration to elicit oxidative stress. The treatment with H_2_O_2_ induced slight translocation of Nrf-2 into the nucleus while treatment with *L. reuteri* elicited marked translocation of Nrf-2. In TR146 cells treated with H_2_O_2_ and combination of *L. reuteri* strains, the expression and amount of nuclear translocated Nrf-2 were greater compared to TR146 cells treated with either H_2_O_2_ or *L. reuteri* mixture alone. The examination of anti-oxidant genes showed that the TR146 cells treated with both H_2_O_2_ and *L. reuteri* demonstrated significant increase in SOD-1, GPx-1 and HO-1 (Fig. 6A–C). To validate that the expression of these anti-oxidant defense genes were under the control of Nrf-2, the expressions of these genes were analysed in TR146 cells lacking Nrf-2 (Supplementary Fig. 1). Indeed, the expressions of SOD-1, GPx-1 and HO-1 did not change significantly following treatment with H_2_O_2_ and *L. reuteri*. To confirm that these *L. reuteri* strains mitigated oxidative stress at the cellular level, the amounts of reduced and oxidized glutathione in TR146 cells were determined. TR146 cells treated at a non-cytotoxic dose of H_2_O_2_ demonstrated significant reduction in GSH/GSSG ratio, indicating oxidative stress (Fig. 6D). However, in cells treated with *L. reuteri*, oxidative stress induced by H_2_O_2_ was alleviated, as demonstrated by an improvement of the GSH/GSSG redox.

## Discussion

There is a growing interest in the applicability of probiotics in OM; largely driven by the effectiveness of probiotics in reducing the severity of intestinal mucositis^18,19^. In this study we showed for the first time that *L. reuteri* DSM 17938 and PTA 5289 significantly reduced 5-FU induced damage to the oral mucosa via the reduction of oxidative stress and inflammation in pre-clinical rodent models. While probiotics are generally considered harmless and are regarded equivalent to food-grade organisms, human infections caused by *Lactobacillus* spp. have been reported^20–22^. This can occur when the probiotic strains possess the ability to translocate systemically causing sepsis and trigger exacerbated immune responses, or harbour mobile genetic elements that allow the transfer of virulence or resistance genes to other bacteria population^23^. Currently, there are no guidelines on the safety of probiotics for patients undergoing anti-neoplastic therapy. Our study found that the oral intake of *L. reuteri* DSM 17938 and PTA 5289 strains in pre-clinical rodent models did not result in systemic bacterial translocation suggesting that these *L. reuteri* strains are safe for administration during chemotherapy.

Currently, there are a handful of clinical studies that have evaluated probiotics use in OM. *Lactobacillus brevis* CD2 lozenges was found to reduce the incidence of grades 3 and 4 OM, and increase the completion rates of anti-neoplastic therapy^19^. In another study, a probiotic combination of *Bifidobacterium longum*, *Lactobacillus lactis*, *Enterococcus faecium* decreased the incidence of grade 3 OM in nasopharyngeal carcinoma patients undergoing chemo- and radiotherapy^18^. However, a recent report using *L. brevis* CD2 found that the probiotic strain did not ameliorate OM severity in head and neck cancer patients undergoing chemo- and radiotherapy^24^. Yet, none of these studies explored the mechanistic actions of probiotics in OM pathogenesis. As different probiotics species/strains could differ in their mode of action in disease processes, understanding the mechanism of probiotics in OM pathogenesis is a key step to its successful clinical adoption in OM management.

In this study, 5-FU, a common anti-neoplastic drug was used to induce OM^25^. The primary mechanism of 5-FU’s action is through complexing with thymidylate synthase, an essential enzyme in DNA biosynthesis which disrupts DNA synthesis, thus triggering the production of highly reactive hydroxyl and superoxide radicals^26^. These ROS induce cell injury and death which manifests clinically as erythema of mucosa and painful ulcers during OM. ROS may also further induce the production of pro-inflammatory cytokines which adds to the cellular oxidative stress^27^. We found that *L. reuteri* DSM 17938 and ATCC PTA 5289 strains significantly reduced 5-FU induced cellular oxidative stress and inflammation to the oral mucosa via the reduction of oxidative stress and inflammation. We further showed that in oral mucosa cells engineered to lack Nrf-2, these *L. reuteri* strains failed to induce the synthesis of anti-oxidant genes compared to the wild-type cells. Collectively, these findings indicate that *L. reuteri* DSM 17938 and ATCC PTA 5289 activated anti-oxidant genes through Nrf-2, a transcription factor in the cap “n” collar (CNC) subfamily of basic-region leucine zipper (bZIP) transcription factors. Under basal condition, Nrf-2 undergoes rapid ubiquination and proteasome degradation^16^. During oxidative stress, Nrf-2 is released from its cytosolic repressor Keap1 and subsequently translocates into the nucleus where it binds to the cis-enhancer element referred to as anti-oxidant response elements (AREs). ARE is found in the promoter region of numerous anti-oxidant genes. We found that the mixture of *L. reuteri* DSM 17938 and ATCC PTA 5289 triggered the nuclear translocation of Nrf-2 in oral mucosa cells. The activation of the Nrf-2 signalling during oxidative stress has been reported in the literature for other *Lactobacillus* spp.^16,28^. While the activation of the Nrf-2 signalling pathway is typically associated with the upregulation of anti-oxidant genes, we found that the translocation of Nrf-2 elicited by *L. reuteri* did not translate to an anticipated increase in anti-oxidant genes. Instead, the anti-oxidant genes expression by *L. reuteri* was triggered only in the presence of oxidative stress, suggesting that perhaps the recruitment of other binding partners and/or co-activators such as c-Jun and activating family of transcription factors (ATF) to the ARE site is required to initiate gene transcription^29,30^. Future studies will be required to shed light into the molecular basis of this observation. Nevertheless, in the presence of cellular oxidative stress, *L. reuteri*’s ability to initiate cytoprotective responses is desirable in OM induced by cancer therapy as the excessive activation of Nrf-2 may promote cancer development and progression by inhibition of cell apoptosis and enhanced survival of oxidative damaged cancer cells^31,32^.

In this study, we found that *L. reuteri* restored ROS-mediated disruptions of glutathione (GSH) levels, re-establishing cellular GSH homeostasis in oral mucosa cells. GSH is the most abundant small molecule thiol in mammalian cells. The functions of GSH include quenching ROS, acting as a co-factor for anti-oxidant enzymes e.g. GPx, regulating cell proliferation, and protecting against apoptosis^33^. Under cellular stress conditions, GSH is oxidized to oxidized glutathione (GSSG). Thus, a decrease in the ratio of intracellular GSH:GSSG redox is indicative of oxidative stress. Other than GSH, anti-oxidant enzymes such as SOD-1, GPx-1 and HO-1 are likewise critical in the detoxification of ROS. GPx-1 which is the most abundant selenoperoxidase present in all cells, catalyses the breakdown of H_2_O_2_. SOD catalyses the dismutation of superoxide radicals, rendering it less toxic; while HO-1 confers cellular protection during stress conditions by increasing the rate of heme catabolism^34^.

*Lactobacillus reuteri* down-regulated 5-FU induced NFκB activation and pro-inflammatory cytokine expression. The down regulation of NFκB may be attributed to *L. reuteri*’s ability to augment Nrf-2 activity. This is consistent with previous reports demonstrating that the activation of Nrf-2 is associated with the attenuation of NFκB responses^35^, and that the depletion of Nrf-2 is associated with enhanced NFκB activation^36,37^. The inhibition of NFκB elicited by these *L. reuteri* strains can occur via direct detoxification of ROS through the upregulation of anti-oxidant defense enzymes, or modulation at the level of transcription factor binding partners specifically through competitive binding for the shared transcription co-activator CREB-binding protein (CBP)^38,39^. Specifically, *L. reuteri* administration prior to initiation of oxidative stress and inflammation (i.e. 5-FU administration) may allow Nrf-2 to preferentially bind to CREB-binding protein (CBP). This subsequently reduces CBP’s availability to be recruited by NFκB; which is activated during the initiation and amplification phases of OM, the end outcome thus is the reduction of NFκB mediated gene expression.

In summary, our study demonstrated that *L. reuteri* DSM 17938 and ATCC PTA 5289 strains protects oral mucosa against damage induced by 5-FU via the activation of cell anti-oxidant defense system and reduction of inflammatory responses. The administration of these *L. reuteri* strains appears to be safe with no evidence of systemic translocation. These findings have led our research group to develop clinical trials to evaluate the efficacy of these probiotic strains as a novel therapeutic agent for cancer therapy induced OM.

## Materials and methods

### Animals and treatment

Ten weeks old female C3H mice were obtained from Taconic Biosciences (Invivos, Singapore). Animals were housed in an environment with temperature of 22 °C, 50% humidity, 12-h light/dark cycle with access to food and water ad libitum*.* The mice were randomly divided into the following experimental groups.

- *PBS/water* Mice injected with phosphate buffered saline (PBS), and fed with normal drinking water (n = 12);
- *PBS/LR* Mice injected with PBS, and fed with the mixtures of *L. reuteri* (LR) DSM 17938 and ATCC PTA 5289 (BioGaia, Sweden) in drinking water (n = 12);
- *5-FU/water* Mice injected with 5-fluorouracil (5-FU), and fed with normal drinking water (n = 12);
- *5-FU/LR* Mice injected with 5-FU and fed with LR in drinking water (n = 12)

Mice were injected with PBS or 50 mg/kg 5-FU intraperitoneally (*i.p.*) daily from days 1 through 5, and on day 8. PBS/water and 5-FU/water groups were given normal drinking water while PBS/LR and 5-FU/LR groups had LR added to the drinking water at 1 × 10^6^ colony forming units (cfu)/mL from day 1. The LR in water was refreshed daily. In addition, the water bottles dosed with LR were positioned at an angle which prevented sedimentation of LR. Mice were sacrificed on day 10. Oral mucosa, blood, spleen and liver were harvested for analysis. To determine if LR translocated systemically, whole spleen or liver were placed in 1 mL sterile 1 X PBS, and homogenized by passing through sterile syringe and needle several times. An aliquot of spleen or liver homogenate, and blood were plated on De Man, Rogosa and Sharpe (MRS) agar, and incubated at 37 °C in the presence of 5% CO_2._ The agar plates were examined for bacterial growth 72 h following incubation. All procedures conformed to the Institutional Animal Care and Use Committee (IACUC) guidelines, and the experimental protocols were approved by the IACUC of the National University of Singapore (protocol number 2015–01106).

### Histopathologic analysis

The oral mucosa tissues were fixed in 10% formalin, dehydrated, and embedded in paraffin blocks. Tissue sections of 5 μm thickness were prepared and stained with hematoxylin and eosin (H&E). Tissues were observed for morphological alterations, and the presence of inflammatory infiltrates under a Leica DMi8 microscope (Leica Microsystems, Wetzlar, Germany). Epithelial thickness was measured using the Image J software version 1.46r (National Institutes of Health, Bethesda, Maryland, USA). All histological examinations were performed in a blinded fashion.

### Immunohistochemistry (IHC) analysis

IHC analysis was performed as described previously^8^. Slides were incubated with primary antibodies against Ki-67 (Thermo Fisher Scientific, MA, USA), 8-OH-dG (Abcam, Cambridge, UK), 4-HNE (Abcam), Nrf-2 (Abcam), NFκB p65 (Cell Signalling Technology, MA, USA), IL-1β (Abcam), TNF-α (Abcam) or MPO (Abcam) overnight at 4 °C. Thereafter, tissues were incubated with biotin-linked secondary antibody followed by signal detection using streptavidin horseradish peroxidase (HRP) conjugate (Thermo Fisher Scientific). Quantitative analysis of the immunolabelled tissues was done in a blinded fashion by counting the number of positively stained cells out of the total number of cells per field at 400 × magnification using Image J software version 1.46r (NIH, Bethesda, Maryland, USA).

### RNA extraction and qPCR analysis

Oral mucosa tissue was homogenized in 1 mL of TRIzol (Thermo Fisher Scientific). Subsequent purification and isolation of RNA was carried out using the GeneAll RNA extraction kit (Seoul, Korea) according to the manufacturer’s protocol. Extracted RNA was treated with DNase I (Promega, WI, USA) to remove residual DNA. RNA was converted into cDNA using iScript reverse transcription Supermix (BioRad, Hercules, CA, USA). qPCR was performed using the CFX Connect Real-Time Detection System (BioRad). A qPCR reaction mixture consisted of 1 μL of cDNA, 10 μL of iTaq Universal SYBR Green Supermix (BioRad), 1 μL each of the respective forward and reverse primers, in a final volume of 20 μL. Thermal cycling was carried out for 40 cycles. The expression of the target gene was determined via the comparative Ct method using β-actin as the housekeeping control gene. PCR primers were designed using the Primer3 software^40^. The sequences of PCR primers used are listed in Table 1.

**Table 1:** Sequences of primers used.

Gene	Species	Sequence	Source
SOD-1	Mouse	Forward 5′-AACCAGTTGTGTTGTCAGGAC-3′	This study
		Reverse 5′-CCACCATGTTTCTTAGAGTGAGG-3′	This study
GPx-1	Mouse	Forward 5′-AGTCCACCGTGTATGCCTTCT-3′	This study
		Reverse 5′-GAGACGCGACATTCTCAATGA-3′	This study
HO-1	Mouse	Forward 5′-AAGCCGAGAATGCTGAGTTCA-3′	This study
		Reverse 5′-GCCGTGTAGATATGGTACAAGGA-3′	This study
β-actin	Mouse	Forward 5′-TCATGAAGTGTGACGTTGACATCCGT-3′	This study
		Reverse 5′-TTGCGGTGCACGATGGAGGGGCCGGA-3′	This study
SOD-1	Human	Forward 5′-GAGCAGAAGGAAAGTAATGG-3′	This study
		Reverse 5′-GATTAAAGTGAGGACCTG C-3′	This study
GPx-1	Human	Forward 5′-CTACTTATCGAGAATGTGGC-3′	This study
		Reverse 5′-CAGAATCTCTTCGTTCTTGG-3′	This study
HO-1	Human	Forward 5′-TCCGATGGGTCCTTACACTC-3′	This study
		Reverse 5′-TAAGGAAGCCAGCCAAGAA-3′	This study
β-actin	Human	Forward 5′-AAACTGGAACGGTGAAGGTG-3′	This study
		Reverse 5′-AGAGAAGTGGGGTGCTTTT-3′	This study

### Cell culture and treatment

The human squamous cell carcinoma cell line TR146 cells was obtained from the European Collection of Authenticated Cell Cultures (Salisbury, UK). The cells were cultured in DMEM (Hyclone, UT, USA) supplemented with 10% fetal bovine serum (FBS) (Hyclone). The cells were incubated in a humidified atmosphere with 5% CO_2_ at 37 °C. TR146 cells were seeded at a density of 0.5 × 10^6^ cells/mL in a 24-well plate and allowed to adhere overnight. An overnight culture of *L. reuteri* was cultured by inoculating an isolated colony into MRS broth (Acumedia, MI, USA), and incubated in a 5% CO_2_ at 37 °C overnight. On the following day, TR146 were either left untreated, pre-treated with the mixtures of *L. reuteri* strains DSM 17938 and ATCC PTA 5289, at a multiplicity of infection (MOI) of 1:1 for both LR strains, for 1 h prior to treatment with 50 μM H_2_O_2_ (Sigma) for 3 h. Total RNA was extracted from TR146 cells using RNAeasy Mini Kit (Qiagen, CA, USA). Subsequently, the expression of target genes were determined by qPCR as described above.

### Analysis of Nrf-2 nuclear translocation

TR146 cells were treated as described above. Cells were fixed with 4% paraformaldehyde followed by permeabilisation with 0.02% Triton-X for 20 min. After washing with PBS, the cells were blocked with 5% bovine serum albumin (Sigma-Aldrich, Missouri, USA) for 1 h at room temperature, followed by incubation with Nrf-2 antibody (Abcam) overnight at 4 °C. Subsequently, the wells were washed and incubated with goat anti-rabbit IgG-Alexa Flour 488 (Abcam) for 1 h and counterstained with 4′6-diamidino-2-phenylindole, DAPI (Abcam) and Phalloidin-iFlour 594 reagent (Abcam) for 5 min each. Stained wells were analysed using a Leica DMi8 fluorescent microscope.

### Nrf-2 gene knockout

A previously validated Nrf-2 CRISPR gRNA target sequence (5′-TGCATACCGTCTAAATCAAC)^41^ was used for CRISPR/Cas9 genome editing. This gRNA cloned into the pLentiCRISPPR v2 plasmid was obtained from GenScript (NJ, USA). TR146 cells were either mock transfected or transfected with Nrf-2 targeting construct with Lipofectamine 3000 (Thermo Fisher Scientific). The transfected cells were either left untreated or treated with H_2_O_2_ and/or *L. reuteri* as described above. The expression of target genes were determined by qPCR as described above.

### Statistical analysis

Statistical analysis was performed using the GraphPad Prism software version 6 (CA, USA). The results were presented as mean ± SD. For data involving two experimental groups, Student’s t-test was carried out. For multiple group analysis, ANOVA was used. Differences with *p *value < 0.05 were considered statistically significant.

## Supplementary information

Supplementary Information.
