# Supplementary material for: Lactobacillus reuteri DSM 17938 and ATCC PTA 5289 ameliorates chemotherapy-induced oral mucositis
Source: Sci Rep. 2020 Oct 1;10:16189. doi: 10.1038/s41598-020-73292-w (PMC7530769; doi:10.1038/s41598-020-73292-w)
Supplement: Supplementary file 1 — Supplementary Information. [file 41598_2020_73292_MOESM1_ESM.docx]

***Lactobacillus reuteri* DSM 17938 and ATCC PTA 5289**

**Ameliorates Chemotherapy-Induced Oral Mucositis**

Nitasha Gupta^1^, Joao Ferreira^1, 2^, Catherine Hsu Ling Hong^1*^ and Kai Soo Tan^1*^

^1^Faculty of Dentistry, National University of Singapore, Singapore

^2^Exocrine Gland Biology and Regeneration Research Group,

Faculty of Dentistry, Chulalongkorn University, Thailand

^*^Corresponding authors:

Kai Soo Tan

Faculty of Dentistry

National University of Singapore

9 Lower Kent Ridge Road,

National University Centre for Oral Health

Singapore 119085

Email: [denkst@nus.edu.sg](mailto:denkst@nus.edu.sg)

Tel: +65-6772-8842

Fax: +65-6778-5742

Catherine Hsu Ling Hong

Faculty of Dentistry

National University of Singapore

9 Lower Kent Ridge Road,

National University Centre for Oral Health

Singapore 119085

E-mail: [denchhl@nus.edu.sg](mailto:denchhl@nus.edu.sg)

Tel: +65-6772-5555 (Ext 11787)

Fax: +65-6778-5742

**Supplementary Figure 1**


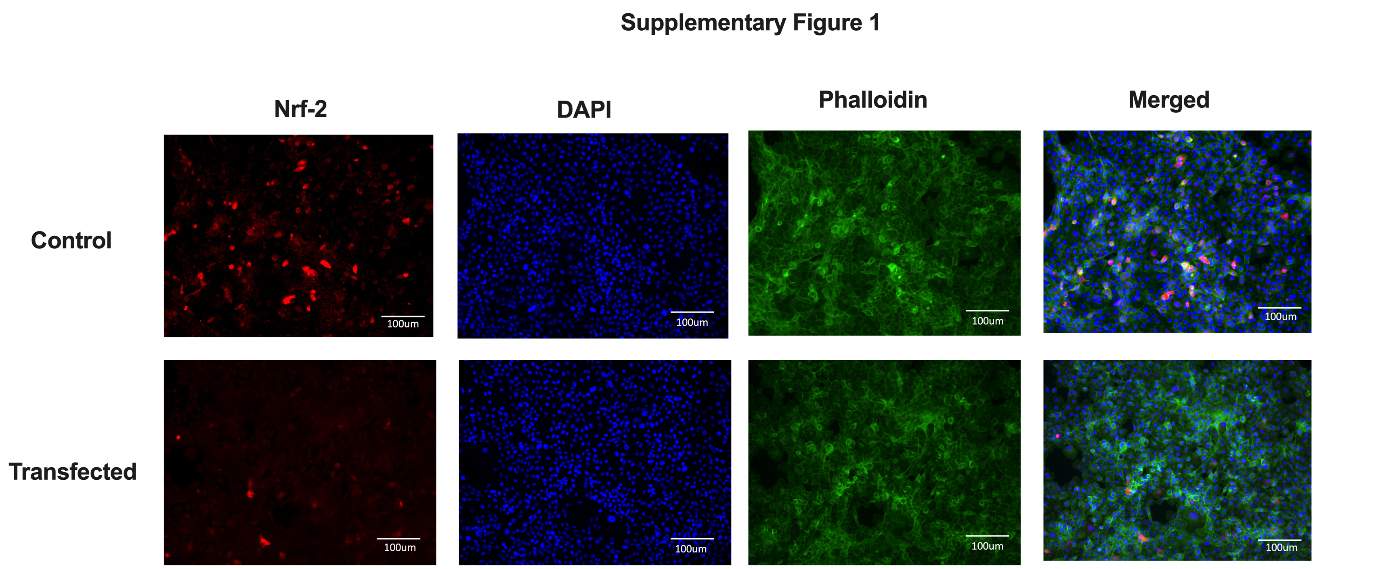


Supplementary Figure 1. Expression of Nrf-2 in TR146 cells. TR146 cells were mock transfected (control) or transfected with pLentiCRISPPR v2 plasmid expressing Nrf-2 gRNA. At 72-hour post-transfection, the expression of Nrf-2 was visualized by immunofluorescence. Nrf-2 was stained red, DAPI stained the nucleus blue while phalloidin stained the actin green. 200x magnification. Representative immunofluorescence images are shown.
